# Molecular Phylogeny of *Burkholderia pseudomallei* from a Remote Region of Papua New Guinea

**DOI:** 10.1371/journal.pone.0018343

**Published:** 2011-03-31

**Authors:** Anthony Baker, Talima Pearson, Erin P. Price, Julia Dale, Paul Keim, Heidie Hornstra, Andrew Greenhill, Gabriel Padilla, Jeffrey Warner

**Affiliations:** 1 Environmental and Public Health Microbiology Research Group, School of Veterinary and Biomedical Sciences, James Cook University, Townsville, Australia; 2 Center for Microbial Genetics and Genomics, Northern Arizona University, Flagstaff, Arizona, United States of America; 3 Infection and Immunity, Papua New Guinea Institute of Medical Research, Goroka, Papua New Guinea; National Institute of Allergy and Infectious Diseases, National Institutes of Health, United States of America

## Abstract

**Background:**

The island of New Guinea is located midway between the world's two major melioidosis endemic regions of Australia and Southeast Asia. Previous studies in Papua New Guinea have demonstrated autochthonous melioidosis in Balimo, Western province. In contrast to other regions of endemicity, isolates recovered from both environmental and clinical sources demonstrate narrow genetic diversity over large spatial and temporal scales.

**Methodology/Principal Findings:**

We employed molecular typing techniques to determine the phylogenetic relationships of these isolates to each other and to others worldwide to aid in understanding the origins of the Papua New Guinean isolates. Multi-locus sequence typing of the 39 isolates resolved three unique sequence types. Phylogenetic reconstruction and *Structure* analysis determined that all isolates were genetically closer to those from Australia than those from Southeast Asia. Gene cluster analysis however, identified a *Yersinia*-like fimbrial gene cluster predominantly found among *Burkholderia pseudomallei* derived from Southeast Asia. Higher resolution VNTR typing and phylogenetic reconstruction of the Balimo isolates resolved 24 genotypes with long branch lengths. These findings are congruent with long term persistence in the region and a high level of environmental stability.

**Conclusions/Significance:**

Given that anthropogenic influence has been hypothesized as a mechanism for the dispersal of *B. pseudomallei*, these findings correlate with limited movement of the indigenous people in the region. The palaeogeographical and anthropogenic history of Australasia and the results from this study indicate that New Guinea is an important region for the further study of *B. pseudomallei* origins and dissemination.

## Introduction

Melioidosis is a severe disease caused by *Burkholderia pseudomallei*, a saprophytic Gram-negative β-Proteobacteria frequently isolated from soil and water in tropical and subtropical regions worldwide [1]. A feature of the disease is spatial clustering of clinical incidence linked to environmental prevalence of the organism. Whilst it is well established that infection follows environmental exposure, factors contributing to the environmental persistence and dispersal of *B. pseudomallei* remain to be elucidated. Molecular typing techniques have allowed detailed phylogenetic studies. Multi-locus sequence typing (MLST) has been used to examine the diversity of *B. pseudomallei* from various geographical regions [2], [3], [4], [5] and revealed patterns of geographical partitioning between Australian and Southeast Asian isolates. Also, it has been determined that Australian isolates are more likely to carry an ancestral *B. thailandensis*-like flagellum and chemotaxis gene cluster (BTFC), whilst isolates from Asia almost exclusively carry a *Yersinia*-like fimbrial gene cluster (YLF) [6]. This suggests that these populations are genetically distinct due to broad scale biogeographical factors associated with establishment and persistence of the organism. More recently, whole genome sequencing has resolved that Asian isolates of *B. pseudomallei* share an Australian ancestral root [7].

Recent studies from Papua New Guinea (PNG) have identified a focus of melioidosis endemicity in a rural community in the Western province. A feature of these isolates is their narrow genetic diversity over spatial and temporal scales [8]. The island of New Guinea is positioned midway between the world's two major melioidosis endemic regions; Northern Australia and Southeast Asia. Detailed phylogenetic study of isolates from this region will advance understanding of the biogeography of melioidosis. This will provide insight into the ecology of *B. pseudomallei* through elucidation of the mechanisms involved in evolution, speciation and dispersal, and assist predictive mapping of disease prevalence. This study employed molecular typing techniques in an attempt to elucidate the origins of the PNG isolates and therefore the role of New Guinea in the dispersal of *B. pseudomallei*.

## Methods

### Ethics Statement

Approval and ethical clearance for this study was granted by the Medical Research Advisory Committee (MRAC) of Papua New Guinea under MRAC No 10.03. All clinical isolates collected originate from diagnostic specimens, and as such patients did not provide written informed. MRAC is the appropriate body in PNG to grant approval for the later use of clinical samples in research, as was the case for this study.

### Bacterial isolates and DNA extraction

**Figure 1:**
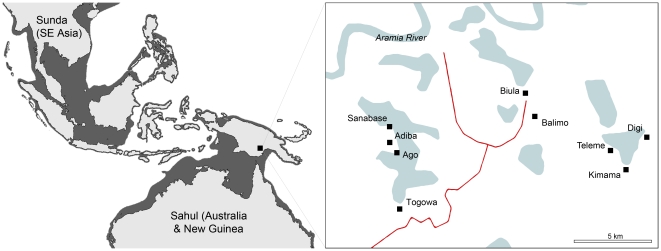
Map of Australasia. **a**) 21,500 years ago during the last glacial maximum. The shaded regions represent what was dry land during the period. Note that Australia and PNG comprised a single continent (Sahul) and that most of Southeast Asia (Sunda) was linked by land bridges [19]. **b**) The Balimo region of the Western Province of Papua New Guinea on the Aramia River floodplain.

This study analyzed 13 clinical (from eight patients) and 26 environmental isolates of *B. pseudomallei* (Table 1), which were retrieved from the Balimo region of PNG (Figure 1) as previously described [8], [9]. Bacteria were stored in the James Cook University culture collection at −80°C in double strength Luria-Bertani broth supplemented with an equal volume of glycerol. Isolates were plated onto Ashdown's agar [10] and cultivated at 37°C for 48 hours prior to DNA extraction. A single colony from each plate was removed for DNA extraction using the RBC Genomic DNA extraction kit (RBC Bioscience, Chung Ho City, Taiwan) as per the manufacturer's directions.

**Table 1:** *B. pseudomallei* isolates used in this study.

ID	Location	Year	Source	MLST
A67	Sanabase	2005	Environmental	667
A78	Sanabase	1998	Environmental	267
AG38	Sanabase	1998	Environmental	267
AG55	Ago	2001	Environmental	667
AG57	Ago	2001	Environmental	667
A02	Ago	2001	Environmental	667
B03	Buila	2005	Environmental	667
BP1	Buila	2005	Environmental	667
C1	Adiba	1995	Clinical	267
C2 & C3	Teleme	1995	Clinical	267
C4	Digi Point	1995	Clinical	267
C5 & C6	Balimo	1998	Clinical	267
C7 & C8	Balimo	1998	Clinical	267
C9 & C10	Togowa	1998	Clinical	267
C11 & C13	Balimo	NA	Clinical	267
C12	Balimo	NA	Clinical	668
K113	Digi Point	1998	Environmental	267
K141	Digi Point	1998	Environmental	267
K24	Teleme	2001	Environmental	267
K33	Teleme	1998	Environmental	267
K41	Teleme	1998	Environmental	267
K42	Kimama	2001	Environmental	267
K93	Kimama	1998	Environmental	267
SA12	Sanabase	2001	Environmental	267
SA15	Sanabase	2001	Environmental	267
SA16	Sanabase	2001	Environmental	267
SA17a	Sanabase	2001	Environmental	267
SA20	Sanabase	2001	Environmental	267
SA24	Sanabase	2001	Environmental	267
SA46	Sanabase	2001	Environmental	267
SA47	Sanabase	2001	Environmental	267
SA48	Sanabase	2001	Environmental	267
SA59	Sanabase	2001	Environmental	267
SA61a	Sanabase	2001	Environmental	267

Multiple identifiers indicate isolates that were isolated concurrently from a single patient. Isolates C5 & C6 and C7 & C8 were recovered from siblings.

### Genotyping using MLST and MLVA

PCRs for MLST were performed using standard reagents from RBC and contained: 50 ng template DNA, 1 × buffer (containing 2 mM MgCl and BSA), 0.8 µM mixed primers, 0.2 mM dNTPs, 0.25 U *Taq* polymerase, and molecular biology grade H_2_O (to 30 µl). Primers for MLST were as described [3] with the recommended amendments listed on the *B. pseudomallei* MLST website (http://bpseudomallei.mlst.net). Cycling conditions consisted of an initial denaturation period of 3 min at 95°C, followed by 40 cycles of 95°C for 30 sec, 62°C for 30 sec and 72°C for 30 sec and a final elongation of 72°C for 10 min. Sequencing products were analyzed by electrophoresis using a 1.5% agarose gel to ascertain correct fragment size, concentration and purity. Sequencing reactions generating insufficient or multiple products were discarded and repeated. Reactions were purified and sequenced by Macrogen (Seoul, South Korea), using the ABI PRISM3700 automated sequencing instrumentation (Applied Biosystems, Foster City, CA). New alleles and sequence types (STs) were submitted to the *Burkholderia* MLST website curator.

Phylogenetic reconstructions were performed using the maximum likelihood algorithm provided by PhyML [11] with the Tamura-Nei nucleotide substitution model against the entire MLST database (http://bpseudomallei.mlst.net), which as of September 2010 encompasses almost 700 distinct STs from more than 1,900 isolates and at least three other closely related species. Bootstrapping was performed 50 times, and trees were optimized for topology, rather than branch lengths and rate parameters. Trees were visualized and modeled using FigTree 1.1.2 software (http://tree.bio.ed.ac.uk).

Assignment of STs into either the Australian or Southeast Asian population was achieved using *Structure* 2.2 [12]. *Structure* uses allelic frequency data to identify population structure and assign individuals to populations. We used MLST allelic data downloaded from the *B. pseudomallei* MLST database for *Structure* analyses. Briefly, 100,000 iterations with a burn-in period of 30,000 iterations were used to determine population assignments of STs using the “admixture” model and assuming two populations as previously established [7]. Here, we report the percentage of iterations in which each PNG ST was placed into the Australian population rather than the Southeast Asian population.

Multiple-locus variable number of tandem repeat (MLVA) characterization was carried out as previously described [13]. Amplicons from 23 VNTR loci were used to characterise the 29 isolates. Amplicon sizes for all isolates at all loci were determined by two independent scorers to reduce bias. The Neighbor-Joining algorithm [14] in PAUP 4.0b [15] was used to illustrate patterns of relatedness among samples.

### Fimbrial gene cluster analysis

Detection of BTFC and YLF gene clusters was performed using a multiplex real-time PCR melt procedure as previously described [6]. Briefly, 15 µl PCRs were comprised of RBC reagents and contained: 5 ng template DNA, 1 × buffer (containing 2 mM MgCl and BSA), 0.3 µM of each primer, 0.2 mM dNTPs, 0.25 U *Taq* polymerase, 5 µM SYTO 9 (Invitrogen, Mulgrave, Australia) and molecular grade H_2_O (to 15 µl). Real-time PCR cycling was performed using a Rotor-Gene 6000 (Corbett Robotics, Eight Mile Plains, Australia) apparatus with an initial denaturation period of 2 minutes at 95°C followed by 40 cycles of 95°C for 15 seconds and 60°C for 30 seconds. Melt analysis was performed post-amplification by ramping amplicons from 65°C to 95°C in 0.2°C increments, with fluorescence acquisition on the FAM channel (excitation at 470 nm, detection at 510 nm). Melt peaks at 80°C and 88°C were considered indicative of BTFC and YLF gene clusters, respectively.

### Antimicrobial Sensitivity Testing

Comparative antimicrobial sensitivity testing was performed using the disk diffusion conditions for *Burkholderia cepacia* as per CLSI antimicrobial testing standards (January 2009). Resistance/susceptibility was defined on the basis of the absence or presence of a visible zone of inhibition (ZOI) around the antibiotic disk. The assay was controlled using *Escherichia coli* ATCC 25922. Antibiotics tested were; chloramphenicol (30 µg), meropenem (10 µg), gentamicin (10 µg), amoxycillin/clavulanic acid (30 µg) and tetracycline (30 µg).

## Results

**Figure 2:**
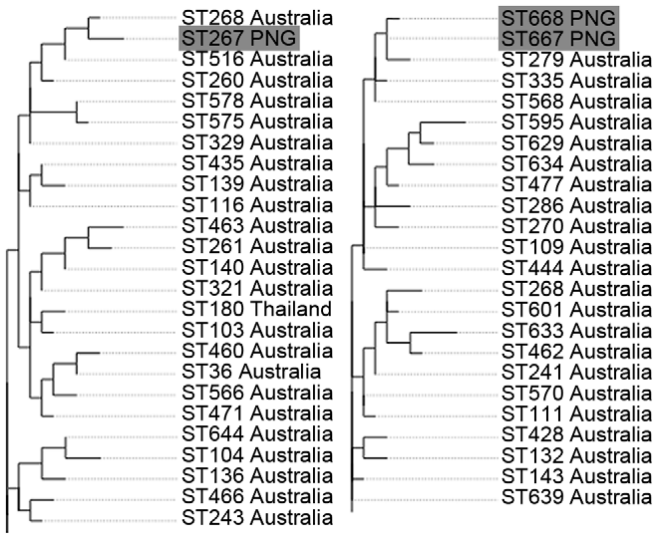
Maximum likelihood tree of *B. pseudomallei* isolates using MLST data. Two individual regions from a maximum likelihood tree constructed using the entire multi-locus sequence typing database showing the relationships of Papua New Guinean *B. pseudomallei* isolates (highlighted in grey) to other sequence types (STs). All three PNG STs fell into regions of the tree heavily dominated by Australian STs.

MLST of the 39 isolates resolved three unique STs. A single ST comprised 32 of the isolates from this area (ST267). The remaining seven Balimo isolates comprised two STs (ST667; *n* = 6 and ST668; *n* = 1), differing from each other by a single nucleotide polymorphism in the *ace* gene, but by four and five locus variations from ST267, respectively. Phylogenetic analysis of the entire *B. pseudomallei* MLST database revealed distinct clustering of Asian isolates (including Malaysian, Singaporean, Philippine and Indonesian STs) separate from those of Australian origin (data not shown). All three STs from Balimo were located in clades heavily dominated by Australian isolates (Figure 2). Population genetic analysis using *Structure* 2.2 also demonstrated that all three STs from Balimo were more closely associated with the Australian population than the Southeast Asian population. ST267 and ST668 were both assigned to the Australian population in 100% of iterations and ST667 was assigned to the Australian population in 99.9% of iterations. Real-time PCR for the fimbrial gene cluster however, determined that all PCR products had distinct melt peaks at 88°C, which is consistent with the YLF type gene cluster found predominantly among isolates of Southeast Asian origin.

**Figure 3:**
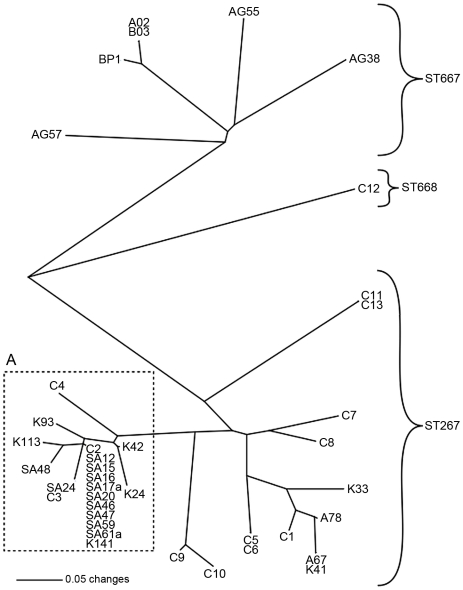
Neighbor joining tree constructed from *B. pseudomallei* MLVA data. The isolates in box A represent those with very short branch lengths and resistance to chloramphenicol, suggesting a high level of relatedness.

To obtain further resolution of the PNG STs, we employed the high-resolution 23-locus MLVA technique to these isolates. MLVA resolved 24 genotypes into three distinct clades, which corresponded with the three different STs (Figure 3). Clinical isolates from the same patient were identical by MLVA on two occasions (C5 & C6 and C11 & C13), but were divergent by one to three loci in an ancestral topography during all other cases (C2 & C3, C7 & C8 and C9 & C10), consistent with *in vivo* mutation as previously reported [16].

All isolates demonstrated susceptibility to meropenem, amoxycillin/clavulanic acid and tetracycline. Resistance to chloramphenicol however, was confined to a single MLVA clade (Figure 3; box A).

## Discussion

Structure and MLST analyses suggest that isolates from the Balimo region are more closely related to those from Australia than Southeast Asia. Although MLST data from *B. pseudomallei* does not provide good phylogenetic resolution due to extremely high rates of lateral gene transfer in relation to mutation, phylogenetic analyses from this and other studies show a tendency for groups of isolates from Australia to cluster independently of Asian isolates. Approaches that compare allele frequencies across populations in order to determine population assignments have been successfully used with MLST data from *B. pseudomallei* and other highly recombinogenic species. Here too, our analyses showed that the three STs identified in Balimo are more closely related to the Australian population rather than the Asian population. Conversely, all of the isolates in this study contained the YLF gene cluster, which is much more common among Asian isolates (98%) than those from Australia (12%) [6]. Given the lower frequency of this gene cluster in members of the Australian population and the close proximity of New Guinea to both Australia and Indonesia, it is possible that *B. pseudomallei* from PNG represent unique genotypes that share characteristics with both major populations.

It has been suggested that geographical ties between PNG and Australia have aided dispersal of the organism during the Last Glacial Maximum (LGM) [7]. The LGM was during the Pleistocene period (approximately 21,500 years ago) at which time sea levels were lower than present, resulting in a continuous land mass from Australia to New Guinea forming the Sahul continent [17], [18]. Similarly, mainland Asia was united with the Indonesian and Philippine islands forming the continent of Sunda, separated from Sahul by the Java and Philippine oceanic trenches [19] (Figure 1a). Concurrently, global temperatures were substantially lower than present, most likely compressing the endemic boundaries of ancient *B. pseudomallei* populations toward the equator. As such, it is likely that human and/or animal movements through New Guinea [20] played a pivotal role in the dissemination of *B. pseudomallei*, however, analysis of a broader range of isolates from the island are required for confirmation.

To elucidate small-scale partitioning of *B. pseudomallei* isolates from Balimo, we employed higher resolution genotyping in the form of MLVA. Studies employing this technique have recently determined that analysis of multiple clinical isolates is necessary due to the high rate of *in vivo* mutation [16], [21]. However, the concept of measuring *in vivo* evolutionary patterns using molecular techniques such as MLVA was in its infancy at the time of sample collection. Despite this shortcoming, the genotyping of this dataset retains its value because the samples typed probably represent the highest frequency *in vivo* genotype. Moreover, the high levels of genetic homogeneity observed among environmental isolates collected over several years (Figure 3; box A) compared to clinical isolates from a single patient collected suggests that both environmental and *in vitro* mutation rates may be limited in comparison to those *in vivo*.

While MLST data suggest low genetic diversity among these isolates, MLVA indicates that genetic diversity exists within the three STs. As only six of the 24 genotypes are represented by more than one isolate, it is likely that our sampling does not reflect the full extent of MLVA diversity. In many cases, branch lengths between isolates are relatively long, reflecting considerable evolutionary divergence. One exception to this is the short branches connecting isolates in box A (Figure 3) suggesting that members of this group (almost half of all the isolates) are closely related to each other. Antimicrobial sensitivity testing bolsters the MLVA in determining that only these isolates are resistant to chloramphenicol; an antibiotic which, due to the limited availability of due to the limited availability of ceftazidime, has been extensively used for the treatment of melioidosis in the region [8]. Given that highly resistant chloramphenicol mutants emerge *in vivo* among 7.1% of patients [22], that chloramphenicol is non-bactericidal [22], and that the excretion of viable *B. pseudomallei* in feces occurs in approximately one quarter of human melioidosis cases [23], it is possible that excretion and cycling of antibiotic resistant genotypes in this subsistent village based community is responsible for the low diversity demonstrated in box A (Figure 3).

The relatively long branches within the other two groups suggest that these lineages of *B. pseudomallei* have persisted in this area for a long period; however, growth rates in the environment and the effects of mutational saturation on branch lengths remains to be determined. Despite this, it is likely, given the eleven clonal isolates in box A (Figure 3), which were collected over six years and from various geographical locations, that this clade represents a stable population of *B. pseudomallei* congruent with our hypothesis of long term stability in the environment, as opposed to recent importation. As only a single isolate from ST668 was collected, it is possible that this ST has not been prevalent in this region for as long as the other STs. Alternatively, as this ST differs from ST667 by just one base substitution at a single MLST locus, it is possible that further sampling will lead to the recovery of more isolates from ST668.

The stability and fragmentation of *B. pseudomallei* populations around Balimo is at least partially impacted by anthropogenic influences. Previously, the introduction of *B. pseudomallei* into non-endemic regions has been linked to the importation of infected animal carriers [24], [25], and anthropogenic influences are highly likely to have been responsible for the dissemination of *B. pseudomallei* from Australia into Asia. Recent studies have estimated that the introduction on *B. pseudomallei* into Asia has occurred within a time-frame congruent with the arrival of the first humans into Austronesia [7]. PNG is highly diverse in terms of culture, language and human genetics due to limited human mobility in the region [26]. If human movement has facilitated dispersal of *B. pseudomallei* between regions of endemicity in other parts of the world, it stands to reason that in regions where human movement is limited, so should the distribution and diversity of *B. pseudomallei* isolates. In conclusion, the role of PNG in the global distribution of *B. pseudomallei* remains to be fully elucidated. Closer analysis of the PNG isolates and phylogenetic reconstructions using a broader range of isolates from the island and adjacent regions such as the Torres Strait will undoubtedly provide a deeper insight into the biogeography of melioidosis.
